# BioCreAtIvE Task 1A: gene mention finding evaluation

**DOI:** 10.1186/1471-2105-6-S1-S2

**Published:** 2005-05-24

**Authors:** Alexander Yeh, Alexander Morgan, Marc Colosimo, Lynette Hirschman

**Affiliations:** 1 The MITRE Corporation, 202 Burlington Road, Bedford, MA 01730, USA

## Abstract

**Background:**

The biological research literature is a major repository of knowledge. As the amount of literature increases, it will get harder to find the information of interest on a particular topic. There has been an increasing amount of work on text mining this literature, but comparing this work is hard because of a lack of standards for making comparisons. To address this, we worked with colleagues at the Protein Design Group, CNB-CSIC, Madrid to develop BioCreAtIvE (Critical Assessment for Information Extraction in Biology), an open common evaluation of systems on a number of biological text mining tasks. We report here on task 1A, which deals with finding mentions of genes and related entities in text. "Finding mentions" is a basic task, which can be used as a building block for other text mining tasks. The task makes use of data and evaluation software provided by the (US) National Center for Biotechnology Information (NCBI).

**Results:**

15 teams took part in task 1A. A number of teams achieved scores over 80% F-measure (balanced precision and recall). The teams that tried to use their task 1A systems to help on other BioCreAtIvE tasks reported mixed results.

**Conclusion:**

The 80% plus F-measure results are good, but still somewhat lag the best scores achieved in some other domains such as newswire, due in part to the complexity and length of gene names, compared to person or organization names in newswire.

## Background

The biological research literature is a major repository of knowledge. Unfortunately, the amount of literature has gotten so large that it is often hard to find the information of interest on a particular topic. There has been an increasing amount of work on text mining for this literature, but currently, there is no way to compare the systems developed because they are run on different data sets to perform different tasks [1]. Challenge evaluations have been successful in making such comparisons. Examples include the ongoing CASP evaluations (Critical Assessment of Techniques for Protein Structure Prediction) for protein structure prediction [2], the series of Message Understanding Conferences (MUCs) for information extraction on newswire text [3], and the ongoing Text Retrieval Conferences (TREC) for information retrieval [4,5]. Also, in 2002, we ran the first challenge evaluation of text mining for biology; this was an evaluation for classifying papers and genes based on whether they contained experimental evidence for gene products [6].

As mentioned in [6], the idea behind these series of open evaluations has been to attract teams to work on a problem by providing them with real (or realistic) training and test data, as well as objective evaluation metrics. These data sets are often hard to obtain, and the open evaluation makes it much easier for groups to build systems and compare performance on a common problem. If many teams are involved, the results are a measure of the state-of-the-art for that task. In addition, when the teams share information about their approaches and the evaluations are repeated over time, then the research community can demonstrate measurable forward progress in a field.

To further the field of biological text mining, the BioCreAtIvE evaluation was run in 2003, with a workshop in March 2004 to discuss the results [7]. The evaluation consisted of two tasks: task 1 focused on extraction of gene names (task 1A) and normalization of genes (task 1B) from PubMed abstracts. Task 2 was a more advanced task focused on functional annotation, using full text information to classify a protein as to its molecular function, biological process and/or location within a cell [8]. This paper reports on task 1A, entity mention extraction. This extraction is a basic text mining operation. Its output is the input text, annotated with the mentions of interest; this can be used as a building block for other tasks, such as task 1B and task 2.

The gene mention task presents a number of difficulties. One difficulty is that gene (or protein) mentions are often English common nouns (as opposed to proper nouns, which, in English, are the nouns normally associated with names) and they are often descriptions. In fact, many entities are named with ordinary words; examples from Drosophila (fruit fly) gene names are *blistery*, *inflated*, *period*, *punt*, *vein*, *dorsal*, *kayak*, *canoe *and *midget*. In addition, new entities are constantly being discovered and/or renamed with these common nouns. Many new names originate as descriptions and can be quite complex, e.g., *hereditary non-polyposis colorectal cancer (hnpcc) tumor suppressor genes*.

### Task and data

The data and evaluation software for task 1A were provided by W. John Wilbur and Lorraine Tanabe at the National Center for Biotechnology Information (NCBI). Every mention of interest is marked, so this task corresponds to the "named entity" task used in the natural language processing community.

The data consists of sentences from Medline [9] abstracts that have been manually annotated for mentions of names of genes and related entities. Half of the sentences were chosen from abstracts likely to contain such names. The other half were chosen from abstracts likely *not *to contain such names. See [10] (also in this volume) for further detail on the construction of the task 1A data. The approximate sizes of the various data sets are given in Table 1.

Participants were provided with 7500 training sentences and 2500 development test sentences. The (final) test set (also known as "round1") consisted of 5000 sentences. For the evaluation, its sentences were renumbered to give no indication of what Medline abstracts they came from (the original sentence numbers were derived in part from the Medline/Pubmed id number of the abstract from which the sentence was drawn).

The data is marked for mentions of "names" related to genes, including binding sites, motifs, domains, proteins, promoters, etc. The data comes with a particular tokenization (word segmentation), and this tokenization determines the boundaries of what is marked. A token is either entirely markable or not. A token *cannot *be split between a marked part and an unmarked part. For example, if "EGF-induced" is a token and one wants to mark the "EGF" part of that token, then one also needs to mark the "induced" part.

For testing, the systems take as input the tokenized unannotated sentences; the output is the list of gene names for each sentence, with the start and stop token offsets. For evaluation, the system output is then compared to the "gold standard" hand-annotated answer key.

There is no detailed, multi-page explicit set of guidelines describing what is markable. Instead, there is a description provided with the data that gives a page or two listing of the types of entities that are and are not markable. Examples of markables are mutants (e.g., *p53 mutant*) and words like *codon *or *antibody *when combined with a gene name. Examples of non-markables include generic terms (e.g., the term *zinc finger *by itself) and mutations (e.g., *p53 mutations*).

Here are 2 excerpts from the training corpus (sentences 110312525757 and 13393732909):

The LMW ***FGF-2*** up-regulated the ***PKC ***epsilon levels by 1.6 fold; by contrast the ***HMW ***isoform down-regulated the level...

...a protein related to ***SNF1*** ***protein*** ***kinase***.

The underlines indicate the markable entities. The ***italic boldface ***indicates what alternative mentions can substitute for a markable. Note that for "SNF1" and "protein kinase", an allowed alternative is "SNF1 protein kinase", which includes both of them.

The answer file for the sentences contains the following mentions: "LMW FGF-2", "PKC epsilon", "HMW isoform", "SNF1" and "protein kinase".

Stored in another file are the alternative mentions that can be tagged and still count as being correct. For the answers mentioned above, here are the allowed alternative mentions: "FGF-2", "PKC", "HMW", and "SNF1 protein kinase".

When scoring, an exact match to an answer or an allowed alternative is needed to get credit for finding an answer. So, for example, if for the answer LMW ***FGF-2***, a system instead returns "The LMW FGF-2", that system would get both a false negative (not matching the answer or its alternative) and also a false positive (the returned item does not match an answer or any alternative).

## Results

15 teams entered submissions for this evaluation. Submissions were classified as either "open" or "closed".   

**Closed: **The system producing the submission is only trained on the task 1A "train" and "(development) test" (devtest) data sets, with no additional lexical resources.   

**Open:** The system producing the submission can make use of external word lists, other data sets, etc.    

Most teams provided an "open/closed" classification for their submissions. If they did not, we made a classification based on a short system description that the teams provided. When we were not sure, we assumed "open".

Teams were allowed to send up to 4 submissions each, as long as they included a "closed" submission. Teams only sending "open" submissions were allowed to send up to 3 submissions. We received a total of 21 "closed" submissions (plus 2 more received late and deemed "unofficial") and 19 "open" submissions (also plus 2 more received late and deemed "unofficial").  

The submissions were measured by their balanced F-score, recall and precision.   

 • Balanced F-score is the harmonic mean between recall and precision.    

Balanced F-score = 2*Recall*Precision/(Recall + Precision)    

• Recall is the fraction or percentage of the answers in the answer key that were found by a submission.    

• Precision is the fraction or percentage of the answers returned by a submission that are scored as correct.  

### Scores achieved by the submissions

Many of the high performing submissions achieved scores quite close together. For example, with balanced F-score, the first and second highest teams were only 0.6% apart, and the second and third highest teams were even closer, at 0.2% apart. This is close enough to possibly be affected by the disagreements in annotation that arise with just about any task on finding entity mentions. An example is that with this particular task, a partial review of the test set changed 0.4% (25 of 6000) of the answers.

These differences are also close enough so that they are often not statistically significant. At a normal threshold of 5% (or lower) for statistical significance, the difference between the first and third highest teams was borderline statistically significant, while the difference between the first and second highest teams (as well as between the second and third highest teams) was not statistically significant.

Computationally-intensive randomization tests [11] (a type of stratified shuffling [12](Sec. 2.7)) were used to test statistical significance. Like most significance tests, 2 variants exist

1. A 1-sided version: under the null hypothesis, how likely will the higher ranked submission be better than the lower ranked submission by at least the observed difference?

2. A 2-sided version: under the null hypothesis, how likely will the difference between the two submissions (in either direction) be at least the observed difference?

The 2-sided version is more demanding and will, for the same score difference, produce about twice the probability of the 1-sided version. Complicating the shuffling done in the tests for these results was the existence of alternative answers, so that the correspondence between correct responses and answers in the key was not one-to-one. Another complication was that due to the format for submissions, no submission could give responses that overlapped with each other.

Comparing the first and third highest teams, the 1-sided test produced a significance level of 4.9%, while the 2-sided produced 9.7% (809 and 1594 out of 16384 trials, respectively). Comparing the first and second highest teams, the 1-sided test was 11% and the 2-sided was 22% (3621 and 7236 out of 32768 trials). Comparing the second and third highest teams, the 1-sided test was 34% and the 2-sided was 68% (351 and 709 out of 1039 trials).

Table 2 shows the high and low scores, as well as the 1^st^, 2^nd ^(median) and 3^rd ^quartile balanced-F, recall and precision scores for the 40 official submissions. Table 3 shows the balanced-F scores of the 40 official plus 4 unofficial submissions. The submissions are grouped by the team that generated the submission, each team is labeled with a letter ("A" through "O") and the teams are sorted by their best F score. One can see a compression or skew of the scores towards the high end.

• The high, 1^st^, 2^nd ^and even 3^rd ^quartile scores are relatively close to each other compared to the low scores

• With F-score, the top 3 teams had F-scores within 1% of each other

• With recall, the top 2 teams were separated by 2.2% in recall

• With precision, the top 2 teams were separated by 0.9% in precision

The difference in recall was statistically significant: 0 out of 3437 trials passed the threshold for either the 1-sided or 2-sided test. The difference in precision was not statistically significant: 6.8% for the 1-sided test and 14% for the 2-sided (1110 and 2310 out of 16384 trials). The F-score differences were discussed above.

Generally, the open submissions did better than the closed submissions. An exception is that for the highest recall score, the top closed score was actually better than the top open score. The compression of the high scores also occurred when comparing the open and closed submissions.

• For the higher scores (like high and 1^st ^quartile), there was little difference (2% or less) between the open and closed submission scores

• For the lower scores (like low and 3^rd ^quartile), the open submissions scores were measurably better than the closed scores

**Figure 1:**
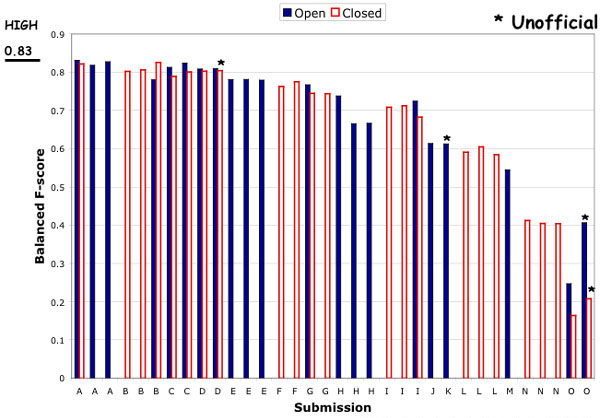
Balanced F-scores of the 40+4 submissions.

Figure 1 shows the balanced-F scores of the 40 official plus 4 unofficial submissions. The open submissions are in a dark solid blue and the closed submissions are in white with a lighter red outline. As in Table 3, the submissions are grouped by the team that generated the submission, each team is labeled with a letter ("A" through "O") and the teams are sorted by their best F score. 13 official submissions from 4 different teams achieved an F-score of 80% or higher (in the figure, this appears as 0.8 or higher). For most teams, their open submission(s) scored higher than their closed submission(s). Team B was an exception, as was team A to some extent. Generally, the gap between a team's open and closed submissions was small compared to the gap between the submissions from different teams. However, team O, which did not do well, had a large gap between their open and closed submissions.

**Figure 2:**
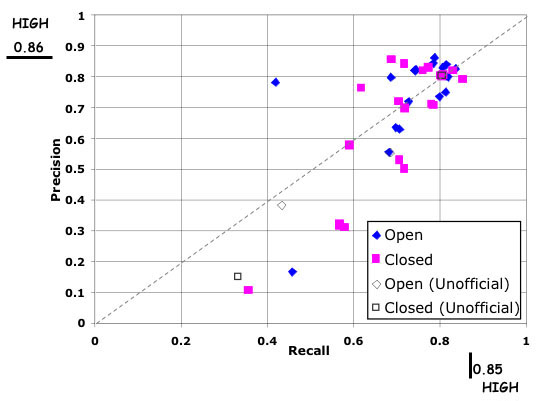
Precision versus recall of the 40+4 submissions.

Figure 2 shows a plot of the precision versus recall of the 40 official plus 4 unofficial submissions. The official open submissions are shown with dark (blue) diamonds, the official closed submissions are shown with lighter (red) squares. Unofficial submissions are shown with gray outlines (and clear centers) of diamonds and squares, respectively. Eight official submissions (from 3 different teams) achieved both a recall and precision of 80% or higher (appears in the figure as 0.8 or higher). As a set, the submissions with both a recall and precision of 60% or more seem to have a fairly balanced precision and recall. But for the most part, submissions which had a recall or precision below 60% tended to have a better recall than precision.

### Some observations

Like many name identification tasks, task 1A has its own unique features. Most teams made use of the training data in their system development. However, in reading the task 1A participants' system descriptions [7], team K did not. Also, as far as we can tell, neither did team M (based on a short description not in the reference). This is probably a reason why, relative to the other teams, these two teams did not get very good results: K's submission had a 61% balanced F-score, while M's submission had 55% (both in the 4^th ^quartile range for official open submissions). One indication of these unique features comes from Tamames [13](Discussion of the results – task 1A), whose system had not considered entities like domains, regions and mutants as "gene names" that should be marked, where as task 1A did include such entities.

A common comment from several task 1A participants (for example, see the post-processing descriptions in Dingare [14](sec. 2.3) and Kinoshita [15](sec. 3)) was that one of the more difficult aspects of task 1A was determining the starting and ending boundaries of the gene-or-protein names. The requirement for an exact match to the answer key (or alternative) increased the difficulty.

As has been mentioned, many of the open and closed submissions achieved fairly close results. One possible reason for this is that, to the extent that this task is unique, outside sources will not help performance that much. When comparing the results between different teams, another possible reason is that for the most part, we relied on the teams themselves to classify their submissions as being "open or closed". In viewing the task 1A system descriptions [7], one can see that the different teams varied in what resources they thought were allowed in a closed submission. As an example, when using a sub-system that generates part-of-speech (POS) tags, some (but not all) teams use such a POS sub-system for a "closed" submission even when the sub-system itself was trained on another annotated corpus, an indirect reliance on an outside corpus. Some teams treated this indirect reliance as permissible for a closed submission (for example, Dingare [14](sec. 2.1) and Zhou [16](sec. 1)), some teams did not.

### Summary of system descriptions

For task 1A, the teams tended to use one of the three following approaches at the top level of their system (see the participants' system descriptions [7]):

1. Some type of Markov modelling.

2. Support vector machine (SVM). Typically, the input information on the word being classified would come from a small window of the words near that word of interest.

3. Rules. As far as we could tell, the rules were usually manually generated.

Many of the systems had pre- and/or post-processing stages in addition to the main approach taken. One system combined several other systems via a voting scheme [16].

The teams used a variety of features in their systems. Many teams used entire sub-systems to find the values of certain features. An example is using a part-of-speech (POS) tagger to find a word's part-of-speech. These sub-systems often used an approach that differed from the overall system's approach.

The four teams with 80% or higher F-scores had post-processing stages in addition to the main approach taken, and made use of many different features. All four of these teams performed some type of Markov modelling at the system's top level [14-17]. However, the teams used different techniques on their Markov models: maximum entropy, hidden Markov models (HMM) and conditional random fields. In addition, one team [16], also had an SVM system at the top level: decisions were made by having two HMMs and an SVM system vote. Also, note that when comparing different systems, the choice of features used is often at least as important as the approach/algorithm used. Yeh [18] gives an example of this.

### Task 1A as a building block

One reason for evaluating on task 1A is that a task 1A system can serve as a building block for other tasks, like task 1B or task 2 of the BioCreAtIvE evaluation. The task 1B evaluation focused on finding the list of the distinct genes (of a particular species) mentioned in a Medline abstract, where the list contained the normalized, unique identifiers for those genes. Task 2 focused on functional annotation (classifying what a protein does and where in a cell it is found), and on returning text passages as evidence to support these classifications.

To what extent was it viable to use task 1A systems as a building block for more advanced capabilities? It turns out that three of the teams taking part in task 1A also took part in task 1B. In addition, one of the three teams also took part in a portion of task 2. So an interesting question is whether these three teams found their task 1A systems to be useful when working on task 1B or 2.

One team (from BioAlma) with a high precision (80%+) task 1A system used the mentions found by their 1A system as the input for their 1B system [13](Task 1B): their 1B system then tried to find the normalized version of the mentions found by their task 1A system.

The story was more complicated for two other teams with both high precision (80%+) and high recall (78%+) task 1A systems. One team was from Pennsylvania (1A: [17], 1B: [19]). The other team was from Edinburgh and Stanford (1A: [14], 1B: [20]). Both these teams looked at some version of finding mentions with their task 1A system and then compared the found mentions against the synonym lists for the genes of interest for task 1B. Both teams found that this approach could easily produce a low precision for 1B, due to ambiguity (many genes sharing many of the same synonyms).

The Pennsylvania team also found that for genes from two (fly and yeast) of the three organisms of interest in task 1B (mouse was the 3^rd ^organism), the task 1A tagger was not that accurate. A possible explanation given was that the task 1A training data did not have enough examples from these two organisms. For task 1B, the Pennsylvania team in the end did not use their task 1A tagger.

The Edinburgh/Stanford team found that using the original task 1A training set and lots of features tended to lower their recall of the 1B genes. They raised the recall by retraining their 1A system using the noisy task 1B training data and a reduced set of the possible features.

The Edinburgh/Stanford team also took part in task 2.1. In this task, a system was given an article, a protein mentioned in that article, and a classification of that protein that a person made based on that article. The system's job was to find a passage of text in that article that supported the classification made for that protein. The description for the team's task 2.1 system [21] made no mention of using their task 1A system or trying it on some part of task 2.1.

## Discussion

### One unique aspect of the data: enforcing a particular tokenization

**Figure 3:**
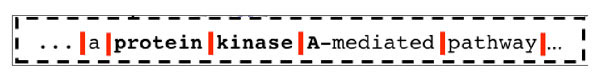
Sample phrase with problematic tokenization (red vertical bars give tokenization boundaries).

As mentioned before, every entity mention task such as task 1A will have some features that are more or less unique to it. For task 1A, one such feature is that the data comes with a particular tokenization (word segmentation). Furthermore, this tokenization affects what counts as a mention, because either all of a token is tagged as part of a mention, or none of that token is tagged. This can cause problems when one just wants to tag part of a token as part of a mention. An example is the phrase "a protein kinase A-mediated pathway", shown in Figure 3, with the red vertical bars indicating the word token boundaries. Here the token "A-mediated" is not useful, since the mention that one would really like to tag is "protein kinase A".

This tokenization is important because it affects what counts as a mention; below are some rules (Lorraine Tanabe, personal communication):

1. If "X" is a token which is a gene name, then "X" is usually marked. An example is "CBF1" in the phrase "... of CBF1 in yeast ..." (in training data's sentence 90233781202).

2. If a token is of the form "X-" or "X-Y", where "X" is a gene name and "Y" is an adjective or verb, then the token is usually NOT marked. An example is "EGF-induced" in the phrase "... block EGF-induced mitogenesis and ... " (in training data's sentence 94547351603).

3. An exception to 2.: when the Y in "X-Y" is "like", then "X-Y" is usually marked (for example, "PRL-like"). Also, if the form is "X-Y Z", where "X-Y" is as in (2), and "Z" is a token like "domain", then "X-Y" is usually marked as part of the mention "X-Y Z". An example is "SH2-binding domain".

### Disagreements in the data

In tasks like task 1A, small disagreements usually exist on what to annotate and what not to annotate. An example in task 1A is phrases of the form "X pathway(s)", where X is a phrase that is marked as part of a gene mention. An initial review of the test set found the following annotation variations (afterwards, all test set cases were changed to have "X" and "X pathway(s)" both allowed as alternative answers):

• 4 cases where "X pathway(s) was NOT an allowed alternative to "X". An example was X = "Mek-Erk1/2" in the phrase

"... the ***Mek-Erk1/2 ***pathway by ..." (sentence 14076).

• 10 cases where "X" and "X pathway(s)" were both allowed alternatives. An example was X = "Ras/Raf/MAPK" in the phrase

"... the ***Ras/Raf/MAPK ***pathway." (sentence 10544).

Similarly, the training set had

• 12 cases where "X pathway(s)" was NOT an allowed alternative to "X".

• 11 cases where "X pathway(s)" and "X" were allowed alternative answers.

Such variation in annotation makes it more difficult to learn or to formulate a rule for how to handle these kinds of constructions.

### Lessons learned for future evaluations

If and when a future task 1A evaluation is run, we list the following issues to consider:

1. Tokenization is non-trivial for biological terms. Perhaps one should *not *enforce a fixed tokenization of the data. This non-enforcement will be expensive because it requires changing both how the data is annotated and how the system results are compared against the gold standard.

2. On a related matter, because of the difficulties in exactly determining a mention's boundaries, there is interest in also counting inexact matches to answers as being correct. This must be defined carefully. For example, if missing either the first or last token still counts as correct, then just returning "epsilon" would count as finding "PKC epsilon".

3. For *open *versus *closed *submissions, we should either remove the distinction, or be more explicit as to what is allowed for a *closed *submission.

4. A suggestion was made to pad the test set with extra material that would not be scored, which would make it harder to "cheat" by manually examining the test set. If this were done, one would need to announce this ahead of time. One reason is that some automated approaches need more processing time than others. Another reason is that some automated approaches, such as transductive support vector machines [22], make use of statistics derived from the entire un-annotated test set.

5. At least one team [14] automatically searched for the PubMed/Medline abstract associated with each test set sentence. They used the abstract as a surrounding context, and it seemed to be helpful. In many "real uses" of a task 1A system, a system will probably have such surrounding text. So it may make sense to just give these abstracts to every participant in the future.

6. There is also a question of what is a permissible resource to use:

• One example is that with PubMed/Medline, a system could also look-up MESH terms, etc. associated with the Medline abstract for each sentence. If a tagging system is applied before an abstract is assigned MESH labels (assignment is done manually), then such information will not be available in real usage, and such information should not be permitted.

• Given a possible entity "X", at least one team [14] did web searches for contexts like "X gene", which support "X" being a possible entity. This seemed to be of limited help. Should this be permitted in the future? This probably depends on the anticipated "real" uses for such a feature. When tagging older material (such as the task 1A test set), the web will have relevant material. When tagging new text that describes new gene(s), the web will probably not have much, if any material.

## Conclusion

For the BioCreAtIvE task 1A of gene mention finding, a number of teams achieved an 80–83% balanced F-score. Unless a system was not performing well, using external resources (being "open" instead of "closed") did not seem to help that much. These 80%+ results are similar to results for some other similar biological mention finding tasks, and are somewhat behind the 90%+ balanced F-scores achieved on English newswire named entity tasks [23].

**Figure 4:**
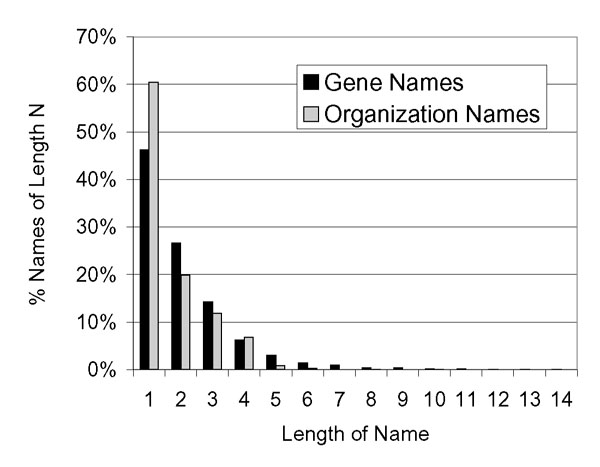
Percent of names of a given length for BioCreAtIvE task 1A gene names and MUC-6 organization names.

Based on an observation offered by Kevin Cohen (associated with the system described in [15]), one hypothesis for the discrepancy is that gene names tend to be longer (in word count) than comparable newswire names. To investigate this, we compared the length distribution of gene names in the test set for task 1A; this distribution is shown in Figure 4, and is compared to the distribution for name length of organization names in a newswire task. The newswire results are computed from the MUC-6 data, which is available from the Linguistic Data Consortium [24]. The average length of the task 1A gene names was 2.09, compared to 1.69 for ORGANIZATION names in the MUC-6 data. Given this distribution, we fitted a simple logistic regression model to both data sets. We modeled the performance (balanced F-score) for a name of *n *words as (*E*)^*n*^, where *E *is the performance on a single-word name. This allowed us to extrapolate back to a single-word error rate for both tasks, allowing us to factor out differences in name length. For gene names, a 91% success rate on a single word gene name gave an overall task performance of 83%, the observed high score. For the MUC-6 organization names, a 95.5% single word success rate yielded a 93% success rate overall, which was the highest recorded result for MUC-6. In using this simple model, we recognize that it is not mathematically valid to use F-measure in place of accuracy. However, it does provide a crude approximation for how much of the task difficulty can be attributed to difference in name lengths among different tasks. This comparison leaves a residual 4–5% discrepancy between performance on the tasks for the single-word case. We hypothesize that this may be due to interannotator variability, leading to "noise" in the training and test data. For the MUC-7 task [25], interannotator agreement was measured at 97%, which is almost certainly significantly higher than for the gene mention task, which has not yet been formally measured. This variability affected at least one high performing task 1A system [26](Discussion). The **Discussion **section of this paper gives some sample disagreements in the task 1A data.

In terms of successful approaches, the teams that achieved an 80% or more balanced F-score tended to use some type of Markov modelling at the top system level. However, these teams also had post-processing stages in addition to the main approach taken, and the different teams made use of different features. These stages and features can have just as much an effect on performance as the main approach taken.

One of the reasons to have task 1A is that it should be a useful building block to work on other tasks, like BioCreAtIvE task 1B. Three teams tried using their task 1A system for task 1B. Their experiences were mixed, with two of the three teams finding that a task 1A system trained on the task 1A training data often did not work so well on task 1B. One of these two teams improved things by retraining their 1A system using the noisy task 1B data.

A 2^nd ^test set is available for task 1A, so it would be straightforward to run a task 1A evaluation in the future using this 2^nd ^test set. Four questions to think about in any future evaluation are the following:

• What will it take to improve task 1A performance?

• How much will improving task 1A performance help with other tasks (like tasks 1B and 2)?

• How can one make a task 1A system be a more useful building block for other tasks?

• Why are outside resources not more useful in task 1A? Is it because task 1A is unique?

## Figures and Tables

**Table 1:** Data set size

Data Set	Sentences	Gene Mentions
training	7500	9000
(development) test	2500	3000
(final) test	5000	6000

**Table 2:** F-score, recall and precision quartiles for the 40 official submissions

	Balanced F-score		Recall		Precision	
	
	open	closed	open	closed	open	closed
High	83%	83%	84%	85%	86%	86%
Quartile 1	81%	80%	81%	79%	83%	81%
Median (Q2)	78%	74%	74%	72%	80%	72%
Quartile 3	67%	59%	70%	62%	72%	53%
Low	25%	16%	42%	36%	17%	11%

**Table 3:** F-score, recall and precision for the 40+4 submissions

Team	Open/closed	precision	recall	balanced-f	unofficial?
A	closed	0.792	0.854	0.822	
A	open	0.828	0.835	0.832	
A	open	0.831	0.805	0.818	
A	open	0.841	0.814	0.827	

B	closed	0.800	0.805	0.802	
B	closed	0.805	0.808	0.806	
B	closed	0.820	0.832	0.826	
B	open	0.751	0.813	0.781	

C	closed	0.819	0.761	0.789	
C	open	0.845	0.784	0.813	
C	closed	0.830	0.773	0.801	
C	open	0.864	0.787	0.824	

D	closed	0.804	0.801	0.803	
D	open	0.803	0.814	0.809	
D	closed	0.803	0.805	0.804	unofficial
D	open	0.801	0.818	0.809	

E	open	0.825	0.742	0.781	
E	open	0.823	0.743	0.781	
E	open	0.823	0.741	0.780	

F	closed	0.855	0.689	0.763	
F	closed	0.843	0.718	0.775	

G	closed	0.712	0.781	0.745	
G	open	0.738	0.799	0.767	
G	closed	0.707	0.785	0.744	

H	open	0.800	0.685	0.738	
H	open	0.637	0.697	0.666	
H	open	0.632	0.705	0.667	

I	closed	0.698	0.719	0.708	
I	closed	0.719	0.706	0.712	
I	closed	0.763	0.617	0.683	
I	open	0.722	0.727	0.724	

J	open	0.558	0.681	0.613	

K	open	0.555	0.683	0.612	unofficial

L	closed	0.501	0.719	0.591	
L	closed	0.529	0.707	0.605	
L	closed	0.578	0.592	0.585	

M	open	0.784	0.418	0.545	

N	closed	0.323	0.568	0.412	
N	closed	0.315	0.567	0.405	
N	closed	0.311	0.579	0.404	

O	closed	0.151	0.332	0.208	unofficial
O	closed	0.107	0.356	0.164	
O	open	0.384	0.432	0.407	unofficial
O	open	0.169	0.457	0.247	
